# Distinct Protein Expression Networks are Activated in Microglia Cells after Stimulation with IFN-γ and IL-4

**DOI:** 10.3390/cells8060580

**Published:** 2019-06-12

**Authors:** Daniele Vergara, Annamaria Nigro, Alessandro Romano, Stefania De Domenico, Marina Damato, Julien Franck, Chiara Coricciati, Maxence Wistorski, Tristan Cardon, Isabelle Fournier, Angelo Quattrini, Michel Salzet, Roberto Furlan, Michele Maffia

**Affiliations:** 1 Department of Biological and Environmental Sciences and Technologies, University of Salento, 73100 Lecce, Italy; daniele.vergara@unisalento.it (D.V.); marina.damato@hotmail.it (M.D.); chiara-crc@libero.it (C.C.); 2 Division of Neuroscience, Institute of Experimental Neurology—IRCCS San Raffaele Scientific Institute, 20132 Milan, Italy; nigro.annamaria@hsr.it (A.N.); romano.alessandro@hsr.it (A.R.); quattrini.angelo@hsr.it (A.Q.); 3 Institute of Food Production Sciences, C.N.R. Unit of Lecce, 73100 Lecce, Italy; stefaniadedomenico@yahoo.it; 4 U1192 INSERM, Laboratoire PRISM: Protéomique, Réponse Inflammatoire, Spectrométrie de Masse, Université de Lille, F-59000 Lille, France; julien.franck@univ-lille.fr (J.F.); maxence.wisztorski@univ-lille.fr (M.W.); cardon.tristan@yahoo.fr (T.C.); isabelle.fournier@univ-lille.fr (I.F.); michel.salzet@univ-lille.fr (M.S.)

**Keywords:** microglia plasticity, microglia, IFN-γ, IL-4, mass spectrometry, proteomics

## Abstract

Microglia cells are the primary immune population of the central nervous system with a role in the regulation of several physiological and pathological conditions. Upon appropriate stimulation, microglia cells can be polarized in a pro-inflammatory M1-like or anti-inflammatory M2-like status. Biological processes and pathways engaged in microglia polarization are starting to be elucidated. To help clarify this, we used a liquid chromatography-mass spectrometry (LC-MS/MS) label free approach to characterize the proteomic profile of human microglia cell line (CHME-5) stimulated with gamma-interferon (IFN-γ) and interleukin-4 (IL-4) to induce a M1 or M2 phenotype, respectively. Outside the classical M1/M2 polarization markers, the M1 status appears to center around the activation of a classical inflammatory response and through the activation of multiple signaling pathways. M2 polarization resulted in a different pattern of protein modulation related to RNA and cellular metabolic processes. Together, our findings provide information regarding the protein changes specific to M1 and M2 activation states, and potentially link the polarization of microglia cells to the acquisition of a specific proteomic profile.

## 1. Introduction

Microglia cells are the tissue-resident immune effector cells of the central nervous system (CNS). Microglia are broadly distributed throughout various regions of the brain [1] and account for 10% of the adult glial cell population within the CNS [2,3]. As primary role, they are actively involved in CNS maintenance with a role in the regulation of neural cell death and survival, neuronal wiring, neuronal activity, and synaptic plasticity [4]. Other important effector functions also include the microglia-mediated control of phagocytosis and the secretion of pro- and anti-inflammatory molecules, thus highlighting microglial heterogeneity and plasticity [5]. Upon disturbance of tissue homeostasis and in response to different stimuli, microglial cells undergo a process of activation that leads to substantial morphological and molecular modifications with both neuroinflammatory and neuroprotective properties [4].

Microglia cells are characterized by a genetic and microRNA profile that significantly differs from tissue macrophages and from other cells of the nervous system [5,6]. They originate from precursors that leave the extraembryonic yolk sac on E8 and migrate through blood vessels into the brain [7], where they are maintained throughout the life by local self-renewal of CNS microglia resident cells [8,9].

In the normal adult CNS, “resting” microglia are high motile cells with a ramified morphology, a small soma with fine cellular processes, and protrusions [10] that physically contact astrocytes, neuronal cell bodies, and blood vessels [11]. Microglia cells can rapidly adapt their functions upon detection of signs of injury that tightly regulate the microglial phenotype. Upon stimulation, resting cells undergo a series of morphological and functional changes with a reaction termed microglial activation. Depending on the environmental milieu and stimulus encountered, different activation status (M1, M2a, M2b, M2c) were proposed [12]. Examples of signals and modulators that regulate microglial activation include abnormal DNA/RNA of bacterial and viral origin, endogenous proteins, complement factors, antibodies, cytokines, chemokines, neurotropic factors, plasma components, neurotransmitters, ions, and other compounds [13]. The full spectrum of the cellular receptors and proteins used by microglia for sensing any changes in the environment represents the microglia sensome that distinguishes these cells from other types of resident macrophages and other cells in the brain [14]. These genes were classified according to gene ontology analysis into pattern recognition receptors (25%), chemoattractant and chemokine receptors (10%), Fc receptors (7%), purinergic receptors (8%), receptors for extracellular matrix (ECM) proteins (6%), cytokine receptors (10%), receptors involved in cell-cell interaction (10%), other receptors or transporters (13%), and potential sensome proteins with no known ligands (11%) [14].

Because of their importance in immune response and possible role in multiple disease states, a deep investigation of the differential proteomic expression in the various microglia activation states is required. To do this, CHME-5 cells, obtained from embryonic fetal human microglia through transformation with simian virus 40 (SV-40) T antigen, were stimulated with interferon-gamma (IFN-γ) and interleukin-4 (IL-4) to induce two different stages of activation, M1-like and M2-like. IFN-γ has been demonstrated to be a critical cytokine for the activation of chemokines, IFN-γ signaling factors, and major histocompatibility complex genes [15], while IL-4 decreases the expression of pro-inflammatory cytokines and fosters microglia to express a developing gene signature [16].

Here, IFN-γ and IL-4 activation stages were characterized by liquid chromatography and mass spectrometry (LC-MS/MS) and subsequent bioinformatics analysis. This led to the identification of specific clusters of differentially expressed proteins that were grouped into enriched biological processes and Kyoto encyclopedia of genes and genomes (KEGG) pathways including immune-related functions, proteasome and ribosome pathways that are characteristic of IFN-γ, and IL-4 treatment.

## 2. Materials and Methods

### 2.1. Cell Culture

The immortalized human microglia cells CHME-5 was obtained directly from the laboratory of Prof. M. Tardieau in Paris, where the cell line was originally created through SV40-dependent immortalization of human fetal microglial cells [17]. Cells were maintained in Dulbecco′s modified Eagle′s medium (DMEM, Euroclone, Milano, Italy) supplemented with 10% fetal bovine serum (FBS), 2 mM glutamine, 100 U/mL penicillin, and 100 μg/mL streptomycin. Cells were cultured in a humidified chamber with a 5% CO2/air mixture at 37 °C. Microglia cells were incubated for 24 h with IFN-γ or IL-4 (20 ng/mL, R&D Systems, Minneapolis, MN, USA) for M1- or M2-like polarization, respectively.

In order to exclude the hypothetical contamination of our cell line with rodent cells as recently reported for the CHME-5 used in numerous laboratories [18,19], CHME-5 cells were tested by flow cytometry with a panel of six different monoclonal antibodies specific for human antigens that did not show cross-reactivity for rodent antigens (as reported in datasheets) (Supplementary Figure S1). To further provide evidence of their human origin, MS/MS spectra were analyzed to identify specific human peptides. A supplementary file containing MS/MS data specific to the human protein interferon-induced protein with tetratricopeptide repeats 1 was reported (Supplementary Figure S2).

### 2.2. Flow Cytometry Analysis

Phenotypical changes in microglia cells were evaluated using a BD Accuri C6 flow cytometer (BD Biosciences, San Jose, CA, USA). Cells were stained with a FITC conjugated-antibody anti-CD40 and an APC conjugated-antibody anti-CD209 (BioLegend, San Diego, CA, USA) diluted in 2% FBS in PBS. Non-specific staining was assessed using isotype controls. Data analysis was performed with FlowJo™ software (v10, Tree Star Inc., Ashland, OR, USA). Results are expressed as ratio between the mean fluorescence intensity (MFI) of the specific antibody and the MFI of the relative isotypic control. MFI ratio values greater than 1 indicate expression of the molecule on the cell surface.

### 2.3. Real-Time PCR Analysis

Total RNA was isolated from CHME-5 cells using RNeasy Mini kit (Qiagen, Hilden, Germany) according to the manufacturer’s recommendations. Isolated RNA was resuspended in nuclease-free water and quantified using a Nanodrop-1000 spectrophotometer (Thermo Fisher Scientific, Rockford, IL, USA). For removal of residual DNA, RNA samples were digested using the Rnase-free DNase Set (Qiagen). Total RNA (1 μg) was retro-transcribed to cDNA using the ThermoScript™ RT-PCR system (Invitrogen, Carlsbad, CA, USA) and random hexamers as primers. The resulting cDNAs were amplified by real-time PCR (CFX96 Touch™ Real-Time PCR Detection System; BioRad, Hercules, CA, USA) using TaqMan Gene Expression Assay (Applied Biosystems, Foster City, CA, USA) for *CD39* (ID: Hs009695559_m1), *CD73* (ID: Hs00159686_m1), *IL10* (ID: Hs00961622_m1), *IL1b* (ID: Hs01555410_m1), *IL6* (ID: Hs00985639_m1), *P2X7* (ID: Hs00175721_m1), *PANX1* (ID: Hs00209790_m1), and *SOD2* (ID: Hs00167309_m1). Human *GAPDH* was used as housekeeping gene (ID: Hs99999905_m1). Relative gene expression was quantified according to the comparative Ct method [20]. Real-Time PCR analysis of fatty acid synthase (*FASN)*, acetyl-CoA carboxylase 1 (*ACACA)*, cluster of differentiation 36 (*CD36)*, diacylglycerol O-acyltransferase 1 (*DGAT1)*, and monoacylglycerol lipase (*MGLL)* was performed as previously described [21]. Results were obtained from three different experiments performed in duplicate and expressed as mean ± SEM.

### 2.4. Western Blot Analysis

Cells were lysed in radioimmunoprecipitation assay (RIPA) buffer (Cell Signaling Technology, Danvers, MA, USA) and proteins were quantified by the Bradford protein assay (BioRad, Hercules, CA, USA). Samples were resolved by SDS–polyacrylamide gel electrophoresis using a Mini-PROTEAN Tetra Cell system (BioRad, Hercules, CA, USA), and transferred to the nitrocellulose membrane (Hybond ECL, GE Healthcare, Chicago, IL, USA). Membranes were blocked for 1 h in 5% nonfat milk and 0.05% Tween-20 (Blotto A, Santa Cruz Biotechnology, Santa Cruz, CA, USA) at room temperature, and subsequently probed by the appropriately diluted primary antibodies for 1 or 2 h at room temperature. Membranes were washed two times for 10 min with a solution containing 10 mM Tris, pH 8.0, 150 mM NaCl, 0.5% Tween 20 (TBST solution), and incubated with a 1:2000 dilution of horseradish peroxidase-conjugated secondary antibodies for 2 h at room temperature. Membranes were washed two times for 5 min with TBST and detected using the Amersham ECL western blotting detection system according to the manufacturer’s protocol (GE Healthcare Life Sciences, Piscataway, NJ, USA). Extracellular signal regulated kinase (Erk1/2) (#4695) and phospho-Erk1/2 (#4370) antibodies were from Cell Signaling and used at the dilution of 1:1000 and 1:2000, respectively. AMP-activated protein kinase (AMPK) (#5831), Src (#2110), phospho-Src (#2101), and phospho-Akt1/2 (Ser473) (#4051) antibodies were provided from Cell Signaling and used at the dilution of 1:1000. Akt1 (sc-5298) antibody was from Santa Cruz Biotechnology (Santa Cruz, CA, USA) and used at the dilution of 1:1000. Phospho-Akt1/2 (Thr308) (#05-802R) antibody was from Millipore (Merck KGaA, Darmstadt, Germany) and used at the dilution of 1:500. P38 (sc-7972) antibody was from Santa Cruz and used at the dilution of 1:2000.

### 2.5. Protein Extraction and Digestion

Protein samples were extracted using the Illustra TriplePrep kit (GE Healthcare Life Sciences, Piscataway, NJ, USA) according to the manufacturer’s instructions and digested according to the filter-aided sample preparation (FASP II) protocol [22]. Briefly, approximately 20 μg of protein extract were dissolved tenfold in a lysis buffer containing 8 M urea in 0.1 M Tris/HCl pH 8.5, loaded into the Microcon Ultracel YM-30 filtration devices (Millipore, Merck KGaA, Darmstadt, Germany), and centrifuged at 14.000× *g* for 15 min. The concentrates were then diluted in 8 M urea and centrifuged again. After centrifugation, proteins were reduced in 10 mM dithiothreitol for 30 min, and then alkylated using 50 mM iodoacetamide for 20 min in the dark. After 4 washes (2 in 8 M urea and 2 in 50 mM NH_4_HCO_3_), trypsin solution was added to the filter at 1:100 (enzyme-to-protein ratio), and samples were incubated at 37 °C overnight in a wet chamber. Digested peptides were collected by centrifugation followed by an additional wash with 50 mM NaCl. Finally, the peptide mixture was acidified by trifluoroacetic acid, desalted-concentrated on C-18 ZipTip (Millipore), dried under vacuum, and then resuspended in 20 µL of acetonitrile/H2O (formic acid 0.1%) (2:98, *v*/*v*).

### 2.6. Mass Spectrometry Analysis, Database Searching, and Bioinformatics Analysis

Peptides were subjected to a reverse phase separation using a nano-EASY-LC 1000 UPLC system (Thermo Fisher Scientific, Rockford, IL, USA) equipped with a 75 μm × 2 cm pre-column with nanoViper fittings (Acclaim pepMap 100, C18, 2 µm, Thermo Fisher Scientific, Rockford, IL, USA) and a 50 μm ID × 150 mm analytical column with nanoViper fittings (Acclaim PepMap RSLC, C18, 2 µm, Thermo Fisher Scientific, Rockford, IL, USA). Peptides were separated using a 2 h gradient of acetonitrile from 5 to 30% over 120 min at a flow rate of 300 nL/min. The Q-Exactive instrument (Thermo Scientific) was set to acquire top 10 MS2 with a spray voltage of 1.6 kV. The survey scans were taken at 70,000 FWHM (at *m*/*z* 400) resolving power in positive ion mode and using a target of 3E6 and default charge state of +2. Unassigned and +1 charge states were rejected, and dynamic exclusion was enabled for 20 s. The scan range was set to 300–1600 *m*/*z*. For the MS2, 1 microscan was obtained at 17,500 FWHM and isolation window of 4.0 *m*/*z*, using a first mass at *m*/*z* 140.

The raw MS data were processed using the MaxQuant software (version 1.5.3.8) [23] and matched to peptide sequences in the human UniProt protein database by the Andromeda algorithm [24]. Trypsin was selected as enzyme and two missed cleavages were allowed. Carbamidomethylation of cysteines was selected as a fixed modification, whereas N-terminal acetylation and methionine oxidation were chosen as variable modifications. For the MS spectra, an initial mass accuracy of 6 ppm was selected, whereas the MS/MS tolerance was set to 20 ppm for the HCD data. False discovery rate (FDR) was set to 1% (peptide and protein level). Relative, label-free quantification of the proteins was done using the MaxLFQ algorithm [25]. The protein group files were imported into Perseus software (version 1.5.2.4) to perform statistical analysis and validation. Hierarchical clustering was performed after z-score normalization of the data within Euclidean distance. Protein–protein interaction (PPI) network and gene ontology (GO) analysis of differentially expressed proteins were generated by STRING version 10.0 (http://string-db.org/). Network enrichment analysis was performed using REACTOME (https://reactome.org/). The transcriptional factor binding site (TFBS) over-representation analysis was performed by InnateDB, using a hypergeometric algorithm and a Benjamini Hochberg correction method as recommended (http://www.innatedb.com/).

## 3. Results

### 3.1. IFN-γ and IL-4 Treated CHME-5 Microglia Cells Presented Distinct M1- and M2-Like Phenotypes

**Figure 1:**
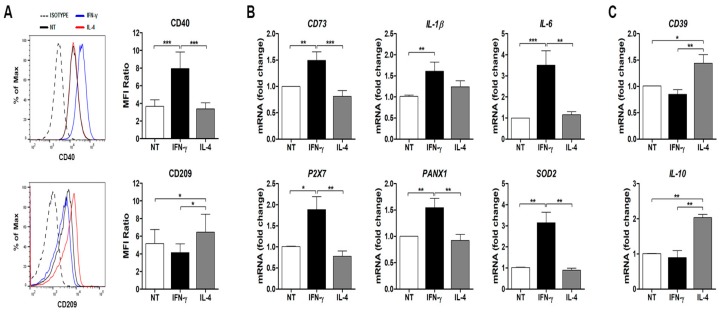
Characterization of the CHME-5 microglia phenotypes stimulated with IFN-γ and IL-4. (**A**) Flow cytometry analysis of CD40 and CD209 surface markers expression. Left panels show representative histogram plots comparing CD40 and CD209 expression by mean fluorescence intensity (MFI) in CHME-5 microglia cells untreated (black line) or treated for 24 h with IFN-γ (blue line) or IL-4 (red line). Right panels report the calculated MFI ratios for CD40 and CD209 expression in untreated (NT), IFN-γ, and IL-4 treated CHME-5 cells. The results represent the mean ± SEM from three independent experiments (* *p* < 0.05 and *** *p* < 0.001; one-way analysis of variance (ANOVA) followed by Bonferroni post hoc test). (**B**,**C**) Gene expression analysis of M1 (CD73, IL-1β, IL-6, P2X7, PANX1, and SOD2) and M2 (CD39 and IL-10) markers by real time PCR in untreated (NT), IFN-γ, and IL-4 treated cells. Results are expressed as mean ± SEM of fold change values relative to the untreated condition (* *p* < 0.05, ** *p* < 0.01, *** *p* < 0.001; one-way ANOVA followed by Bonferroni post hoc test).

Microglia are resident immune cells of the central nervous system (CNS) that change/polarize their phenotype to respond to different processes including inflammation, tissue remodeling, and neurogenesis. Although specific classification of microglia phenotypes remains controversial, microglial activation phenotypes are generally categorized as pro-inflammatory M1 type and anti-inflammatory M2 type similarly to the peripheral monocytes/macrophages phenotypes but with some differences, including the limited value of classical biomarkers to provide a functional significance of these different states [26]. In this context, the focus is now moving onto unbiased approaches, including proteomics, to disclose novel microglia biomarkers associated with specific activation states [26]. Here, to investigate proteome modifications associated with distinct microglial phenotypes, human CHME-5 microglia cells were either unstimulated (M0) or polarized into M1 or M2 phenotype by exposure for 24 h to canonical activation stimuli, namely, IFN-γ for classical M1 activation and IL-4 for alternative M2 activation. Differentiation to M1 or M2 microglia was determined by flow cytometry analysis using M1- (CD40 [27,28]) and M2-specific (CD209 [29,30]) surface markers (Figure 1A). As expected, M1-polarized microglia exhibited an increase in CD40 expression when compared to unstimulated and IL-4 treated cells while microglia stimulated with IL-4 showed specific induction of CD209 expression (Figure 1A). To further validate the phenotype of microglia polarization, a panel of marker genes known to be differentially expressed under either pro-inflammatory (*CD73*, *IL1B*, *IL6*, *P2X7*, *PANX1* and *SOD2*) or anti-inflammatory (*CD39* and *IL10*) conditions was tested by real-time PCR on independent datasets. Accordingly, microglia stimulated with IFN-γ exhibited significant up-regulation of M1-associated genes compared to untreated and M2 polarized microglia (Figure 1B) while IL-4 stimulation induce a significant increase of the M2-specific genes expression (Figure 1C) compared to untreated and IFN-γ-polarized microglia. Overall, these results confirm that the human CHME-5 microglia cells can be polarized into M1- or M2-like phenotype using IFN-γ for classical M1 activation and IL-4 for alternative M2 activation. The same polarization protocol was applied to all the experiments described below.

### 3.2. Characterization of Proteomic Changes in Classically (M1) and Alternatively (M2) Activated CHME-5 Microglia Cells

**Figure 2:**
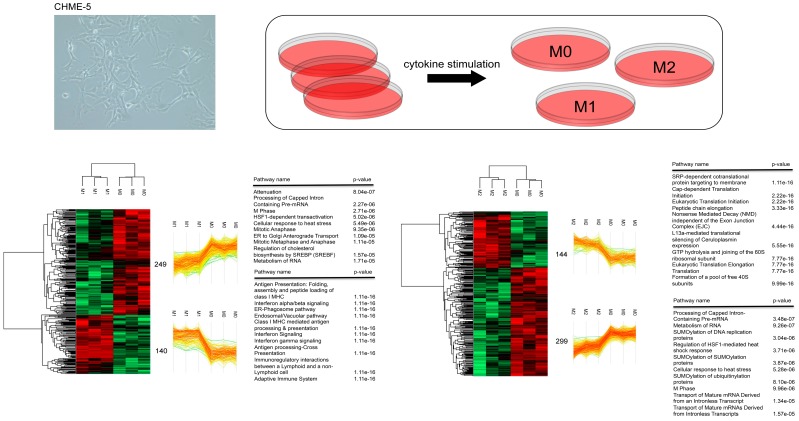
Mass spectrometry analysis of microglia cells. CHME-5 cells were treated with IFN-γ or IL-4 to induce an M1-like or M2-like phenotype, respectively. Heat-map of whole protein abundance profiles following hierarchical clustering based on Euclidean distance of significantly regulated proteins. Three biological replicates are shown for control cells (M0), and for cells stimulated with IFN-γ (M1) and IL-4 (M2). Green color corresponds to a decreased expression level and red color to an increased expression level. Distinct clusters for M0 and M1 samples, and M0 vs. M2 samples are highlighted. Numbers indicate the proteins in each cluster. Proteins in each cluster were analyzed using REACTOME. Top 10 enriched networks identified by functional analysis of differentially expressed proteins and ranked according to *p*-value were visualized.

We analyzed proteins of CHME-5 cells in their basal state (M0) and after stimulation to obtain an unbiased picture of the effects of IFN-γ (M1) and IL-4 (M2) on the microglia proteome. Whole proteins were trypsin-digested by FASP and analyzed by high-resolution MS using a quadrupole Orbitrap instrument. At a peptide and protein FDR of 1%, we identified 2925 proteins across proteome samples from CHME-5 cells (Supplementary Table S1). We performed quantification by a label-free method and determined protein differences by Perseus software analysis. To define proteome changes after microglia cells stimulation, we first identified proteins significantly changed between two groups by *t*-test (*p* <0.05). A heat-map that showed the clusterization of M1 and M2 cells compared to M0 in three biological replicates was generated (Figure 2). For each phenotype, a specific dataset of differentially expressed proteins was identified (Supplementary Table S2), suggesting that the stimulation of CHME-5 with IFN-γ and IL-4 induces at the proteomic level the activation of a specific molecular program. To statistically reveal the presence of enriched biological processes and pathways, protein datasets were subjected to STRING and REACTOME analysis to define protein-protein interaction (PPI) networks and possible enriched terms. Results of pathway analysis performed by REACTOME are presented in Figure 2; the ten most significant terms for the datasets of up- and down-regulated proteins in M1 and M2 groups compared to M0 were shown. We found that networks associated immune functions (i.e., antigen presentation, Interferon alpha/beta signaling, and others) were positively associated with IFN-γ treatment. In this respect, the identification that canonical immune networks are enriched in M1 samples is remarkably consistent with the stimulation of cells with IFN-γ and the activation of a specific gene program mediated by the IFN-γR through the signal transducer and activator of transcription (STAT) signaling. Accordingly, STAT1, STAT2, and STAT3 were identified in the list of up-regulated proteins providing an indication about the role of STAT-dependent pathway in the M1 polarization state. On the other hand, the datasets identified in IL-4-stimulated microglia cells were dominated by the enrichment of networks related to metabolism and RNA and protein processing. These findings are consistent with the view that multiple layers of regulation are involved in the control of microglia polarization [31] and suggest that the activation of gene expression and post-transcriptional regulation mechanisms play a dominant role in regulating protein abundance after IFN-γ and IL-4 stimulation. For instance, SRP-dependent cotranslational protein targeting to membrane, eukaryotic translation initiation, metabolism of RNA, and SUMOylation of SUMOylation proteins were significantly represented in M1 and M2 datasets.

In order to evaluate if possible functional interactions may emerge between these pathways, we identified protein–protein interactions maps by PPI analysis using STRING database (Supplementary Figures S3 and S4). An interconnection between protein groups and the enrichment of specific gene ontology (GO) terms emerged from this analysis. After IFN-γ treatment, type I interferon signaling pathway (17 members, false discovery rate (FDR) 5.23e-19) and RNA processing (16 members, FDR 0.000236) emerged as two distinct and inter-connected groups in the PPI map of M1 up-regulated proteins, indicating the concordant regulation of these modules during microglia activation (Figure S2). On the contrary, no main sub-networks were identified in the PPI map of M1 down-regulated proteins that was dominated by a large module (FDR 1.48e-07) of 141 proteins associated with cellular metabolic processes. We then used gene ontology to characterize proteins modified after IL-4 treatment. We found that biological processes involved in RNA metabolic process (29 members, FDR 6.37e-15) and cellular metabolic process (185 members, FDR 4.43e-16) resulted as being significantly enriched after IL-4 stimulation (Supplementary Figure S4).

Overall, GO analysis of pathways and biological processes revealed that modulation of microglia polarization results from the combination of multiple layers of regulation, including changes in metabolic proteins that may impact on cellular reprogramming by supporting a specific metabolic demand and the production of essential metabolites [32].

**Figure 3:**
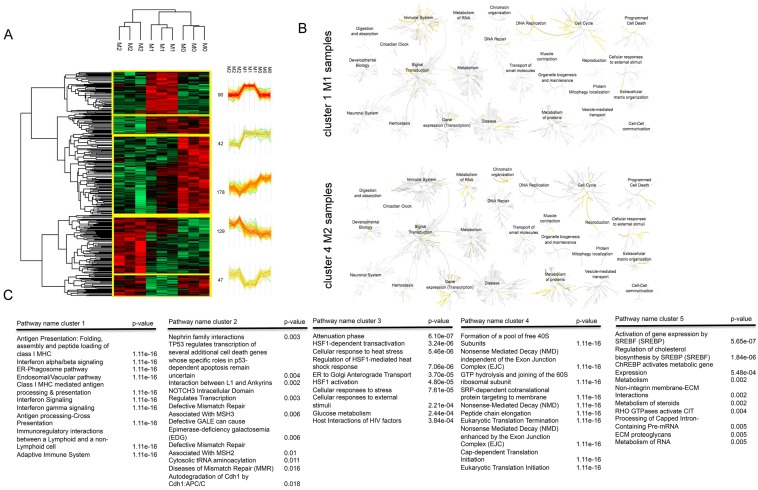
Mass spectrometry analysis of microglia cells. (**A**) Data from three independent biological experiments were combined and analyzed with MaxQuant. Hierarchical heatmap clusters obtained by Perseus of significant proteins from ANOVA across the three groups. Green color corresponds to a decreased expression level and red color to an increased expression level. Profile plots of five clusters showing distinct behavior with respect to the three groups are shown and highlighted in yellow in the heatmap. (**B**) Top 10 enriched networks identified by functional analysis of differentially expressed proteins and ranked according to *p*-value. Data were analyzed using REACTOME. (**C**) Genome-wide overview pathway analysis of cluster 1 and cluster 4 performed using REACTOME. The yellow color code denotes over-representation of that pathway.

To define protein clusters distinctively regulated in IFN-γ- and IL-4-stimulated microglia cells, proteomic data were re-analyzed by analysis of variance (ANOVA) to identify the proteins that are differentially expressed among the three conditions. This analysis provides a summary of the expression profile of each sample and their clear separation in three distinct groups. As shown in hierarchical heat-map of the three groups of samples (Figure 3A), five main clusters were generated. Cluster 1 includes 90 proteins that are up-regulated in the M1 phenotype compared to M0 and M2, cluster 2 contained a group of 42 proteins with a reduced expression in M2 phenotype compared to M0 and M1, cluster 3 includes a group of 178 proteins with a reduced expression in M1 and M2 phenotype compared to M0, cluster 4 includes 129 proteins with a higher expression in the M2 group compared to the other groups, and cluster 5 includes 47 proteins with an increased expression in M0 and M2 phenotype compared to M1. REACTOME analysis revealed significant enriched networks in each cluster of proteins. Top ten networks are showed in Figure 3B. In detail, cluster 1 was significantly enriched of type immune signaling networks, cluster 2 includes networks involved in cell cycle and signaling regulation, cluster 3 was enriched for networks of proteins involved in the cellular response to external stimuli (attenuation phase, HSF-1 dependent transactivation, cellular response to heat stress), cluster 4 includes developmental biology and metabolism of proteins and RNA networks, while cluster 5 was significantly enriched for metabolic networks A genome-wide overview of the results of pathway analysis for cluster 1 and 4 was also reported (Figure 3C). Cluster 1 represents proteins up-regulated in M1 samples compared to M0 and M2, while cluster 4 proteins up-regulated in M2 samples compared to M0 and M1. The graphical representation provides an overview of over-expressed networks and sub-networks (highlighted in yellow) of our datasets. Both clusters represent proteins up-regulated after IFN-γ and IL-4 stimulation, and thus that protein groups that may provide some biological insight concerning the action of the two cytokines. In particular, we observed that GO terms such as immune system, DNA replication, and cell cycle networks emerged from cluster 1 proteins analysis, while chromatin organization and developmental biology were over-represented after IL-4 stimulation, confirming the major functional differences between IFN-γ and IL-4 differentiated microglia cells.

### 3.3. Validation of Signaling and Metabolic Pathways Determined by Proteomics

**Figure 4:**
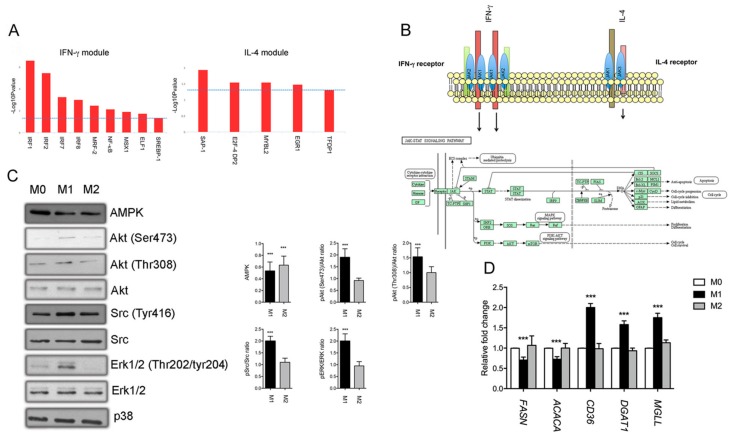
Transcriptional regulation and regulatory signaling pathways activated after exposure of CHME-5 with IFN-γ and IL-4. (**A**) Transcriptional factor binding site (TFBS) over-representation analysis result by InnateDB performed on IFN-γ and IL-4 specific protein clusters identified by ANOVA and represented in Figure 4. (**B**) Schematic representation of IFN-γ and IL-4 receptors and key signaling pathways involved. (**C**) Western blot analysis of Erk1/2, Erk1/2 (Thr202/tyr204), Src, Src (Tyr416), Akt, Akt (Thr308), Akt (Ser473), AMPK in M0, M1, and M2 cells. P38 was used as loading control. The graph shows the relative densitometric quantification of band intensities measured using ImageJ analysis software. Values are means ± S.D. of three independent experiments. *p* value *** < 0.001 compared to M0 cells. (**D**) Differential expression of enzymes involved in lipid metabolism between M0 and M1 cells. *FASN*, *ACACA*, *CD36*, *DGAT1*, and *MGLL* mRNA levels in M0, M1, and M2 cells. Values are means ± S.D. of three independent experiments. *p*-Value *** < 0.001 calculated by ANOVA.

Physiologically, IFN-γ and IL-4 signal through different receptors but both are able to activate the STAT pathway. In this respect, our analysis established a clear correlation between IFN-γ treatment and the activation of STAT with the subsequent over-expression of IFN-responsive genes. However, little is known about other transcription factors (TFs) that may explain the observed proteome differences in polarized microglia cells. We explored the involvement of transcription factors in IFN-γ and IL-4 polarized microglia using the TF binding prediction toll provided by InnateDB database. Over-represented transcription factors binding sites generated using cluster 1 and cluster 4 protein datasets were showed in Figure 4A. For microglia cells stimulated with IFN-γ, analysis included interferon-regulatory factor (IRF) proteins and NF-κB that have been linked to type-I interferon signaling [33]. Other TFs identified in the IL-4 module included proteins that are not previously associated with microglia activation programs. Overall, this analysis highlights a distinct transcription effector program from M1-like and M2-like microglia.

In addition to Janus kinase (JAK)/STAT activation, other kinases can signal downstream the IFN-γ and IL-4 receptors to mediate specific cellular effects, including AMPK, Akt, Src, and Erk kinases [34], as also illustrated in the JAK/STAT KEGG pathway reported in Figure 4B. These kinases may be activated by both cytokines or selectively regulated. To determine if IFN-γ and IL-4 microglia treatment influence the expression and phosphorylation status of these proteins, western blot analysis was performed (Figure 4C). Treatment with IFN-γ resulted in increased levels of phosphorylation of Src at Tyr^416^, Erk1/2 at Thr^202^/Tyr^204^, Akt at Thr^308^, and Ser^473^ in M1 microglia compared to M2 and M0 cells. Moreover, a reduced expression of AMPK was observed in M1 and M2 cells compared to M0. Overall, a distinct signaling signature was observed in microglia cells in response to IFN-γ stimulation. In contrast, no main changes were observed in the phosphorylation status of these kinases after treatment of CHME-5 cells with IL-4 for 24 h. This excludes a sustained effect but not a possible more rapid activation of these kinases by IL-4.

Accumulating evidence suggests that different metabolic pathways are required for programming M1 and M2 macrophages [35,36,37]. At present, the spectrum of metabolic changes necessary to sustain different microglia phenotypes and their functional significance remain less investigated but is supported by data that links metabolic changes to specific polarization states [38]. Our findings highlight that metabolic reprogramming may be important for microglia polarization. This hypothesis was confirmed by MS data and GO analysis, as stated above. To provide a preliminary validation of these data, we interrogated protein clusters reported in Figure 3 searching for metabolic proteins specifically associated with M1 and M2 status. In order to prioritize some specific metabolic pathways, we observed that M1 cells are characterized by changes in the expression of proteins involved in the biosynthesis and metabolism of lipids. For instance, serine beta-lactamase-like protein LACTB, mitochondrial (LACTB), fatty acid synthase (FASN), acetyl-CoA carboxylase 1 (ACACA), and fatty acid desaturase1 (FADS1) and FADS2 were modulated after M1 polarization by suggesting a possible lipid reprogramming in M1 cells. In this respect, we hypothesized that cells with a decreased expression of proteins involved in de novo fatty acids synthesis may increase the uptake of lipids from the microenvironment to sustain their metabolic demand as a functional consequence of an energetic switch [38]. To test this hypothesis, we investigated by real-time PCR the mRNA expression of *FASN* and *ACACA*, *CD36*, diacylglycerol acyltransfearse 1 (*DGAT1*), and monoacylglycerol lipase (*MGLL*) in M0, M1, and M2 cells. The last three genes codify for proteins involved in the uptake of lipids (*CD36*), synthesis of triglycerides (*DAGT1*), and mobilization of fatty acids from triglycerides (*MGLL*). We found that IFN-γ significantly inhibited the expression of *FASN* and *ACACA*, not only affecting their protein levels as demonstrated by proteomics but also regulating gene expression. Concurrently, an increased expression of *CD36*, *DGAT1*, and *MGLL* were observed in CHME-5 cells treated with IFN-γ and not IL-4 (Figure 4D). We speculate that the IFN-γ mediated modulation of genes involved in lipogenesis and lipolysis may divert fatty acids away from anabolic toward catabolic processes.

## 4. Discussion

The physiological and molecular effects induced after IFN-γ and IL-4 treatment are well established and characterized by different techniques in several cellular and animal models [39,40]. These studies support the notion of an IFN-γ and IL-4 associated gene signature that is useful to characterize at the level of single biomarkers, as the dynamic molecular changes the two cytokines [15,16,39,40]. Moreover, data that emerged from these studies also revealed a large diversity in proteins and pathways modulated after treatment, sustaining the application of unbiased approaches to characterize these different functional states [26]. In this study, we attempted to assess the proteome changes induced after stimulation of CHME-5 cells, providing at network-level information about the molecular mechanisms underlying microglia polarization. Microglia cells have a strict control on brain homeostasis, thus a molecular definition of their response to both cytokines will aid in understanding new possible modulators of microglia-mediated inflammation or anti-inflammation status. This approach is conceptually similar to other studies that applied microarray to study gene expression [15], but novel in the application of MS to the study of microglia cells. The integration of all these datasets will be important to provide a complete framework of the mechanisms that at the transcriptomic and proteomic levels are associated with microglia reprogramming. For instance, one of the main findings of this integrative approach was the identification of microglia mRNA and protein networks that may converge or diverge following innate immune challenge [41].

To test how IFN-γ and IL-4 modify the molecular profile of CHME-5 microglia cells, we applied an unbiased shotgun approach combined with a preliminary characterization of the M1/M2-like polarization status of CHME-5 cells and a western blot analysis of signaling pathways activated after stimulation. Besides the list of M1- and M2-associated proteins, data presented are the first large proteomic characterization of this cellular model. Proteins involved in multiple biological processes and molecular functions were identified, including specific markers of microglia identity (beta-hexosaminidase subunit beta) (Supplementary Table S1). This represents a starting point and valuable resource for future studies aimed at investigating changes in protein networks associated with neuroinflammation or neurological disorders.

CHME-5 cells stimulated with IFN-γ and IL-4 activate an M1-like and M2-like polarization status as confirmed by the expression of major markers such as CD40, IL-6, CD73, IL-10, and CD39. The two cytokines bind distinct cytokine receptors, IFN-γR and IL-4R, that signal trough distinct common conventional and unconventional signaling pathways. Common downstream pathways include JAK and STAT, in combination with other kinases. Here, we demonstrated that IFN-γ induces a pro-inflammatory gene expression program by regulating the expression of transcriptional factors STAT1, STAT2, and STAT3, and by increasing the phosphorylation status of Akt, Erk1/2, and Src, raising the hypothesis that these kinases might complement the function of STAT pathway in CHME-5 microglia. Our data, together with previous works demonstrating the activation of these kinases in other CNS models including neurons but not astrocytes [42], support the notion of a cell-specificity with functional physiological effects. For instance, Erk1/2 activation in neurons counteracts STAT1-dependent cell death during IFN-γ exposure. After 24 h of treatment, IFN-γ did not impair CHME-5 viability (data not shown), probably in support of a similar mechanism of action. Moreover, it remains to be determined if the activation of Src/Akt/Erk is mediated by a STAT-dependent or independent pathway. In the MCF-7 epithelial cell model, Src mediates the IFN-γ induced phosphorylation of Erk through H2O2 production [43]. These results led us to hypothesize that Src/Erk activation in microglia cells may respond to a common upstream regulator. Moreover, we observed that AMPK is down-regulated in both conditions, in line with the observation that a reduced AMPK signaling in vivo correlates with increased expression of IFN-γ in the CNS [44]. Overall, these data describe a modulation of Akt, Erk, Src, and AMPK pathways in IFN-γ treated microglia and have implications for inhibitors targeting these proteins to block M1 polarization. However, in the context of networks that we observed modulated by proteomics, the role of these pathways in mediating cell-specific processes remain to be determined. Thus, microglia cells can rely on the activation of multiple signaling pathways to modulate their proteome after IFN-γ stimulation, this complement with the transcriptional, translational, and post-translational layers or regulation that emerged from bioinformatics analysis. Further studies perturbing these proteins can reveal their functional importance. In this scenario, microglia cells stimulated with IFN-γ and IL-4 showed a high degree of plasticity in terms of modulated metabolic proteins. This coupling of metabolism to the anti- or pro-inflammatory phenotype of immune cells has been already described and associated with a specific bioenergetic demand and utilization of different metabolic substrates (glucose, amino acids, or fatty acids) [38]. Congruent with these observations, several enzymes with important immunomodulatory properties were identified in our dataset. Examples include FASN and FADS1 [45,46] that are both required to modulate immune cell plasticity after exposure to pro- or anti-inflammatory stimuli.

In summary, our approach provides a wide view of the protein networks significantly enriched in IFN-γ and IL-4 stimulated microglia cells. This may facilitate the prioritization of therapeutic targets necessary for the acquisition of a pro- and anti-inflammatory condition.

## Supplementary Materials

The following are available online at https://www.mdpi.com/2073-4409/8/6/580/s1, Figure S1: Expression of human cell surface markers in CHME-5 cells, Figure S2: MS/MS analysis of IFIT1 protein, Figure S3: Protein-protein interaction networks of differentially expressed proteins in M0 and M1 groups generated using the STRING database, Figure S4: Protein-protein interaction networks of differentially expressed proteins generated in M0 and M2 groups generated using the STRING database, Table S1: List of identified proteins in CHME-5 cells, Table S2: List of differentially expressed proteins.
