# Supplementary figures and images for: Distinct Protein Expression Networks are Activated in Microglia Cells after Stimulation with IFN-γ and IL-4
Source: Cells. 2019 Jun 12;8(6):580. doi: 10.3390/cells8060580 (PMC6628119; doi:10.3390/cells8060580)

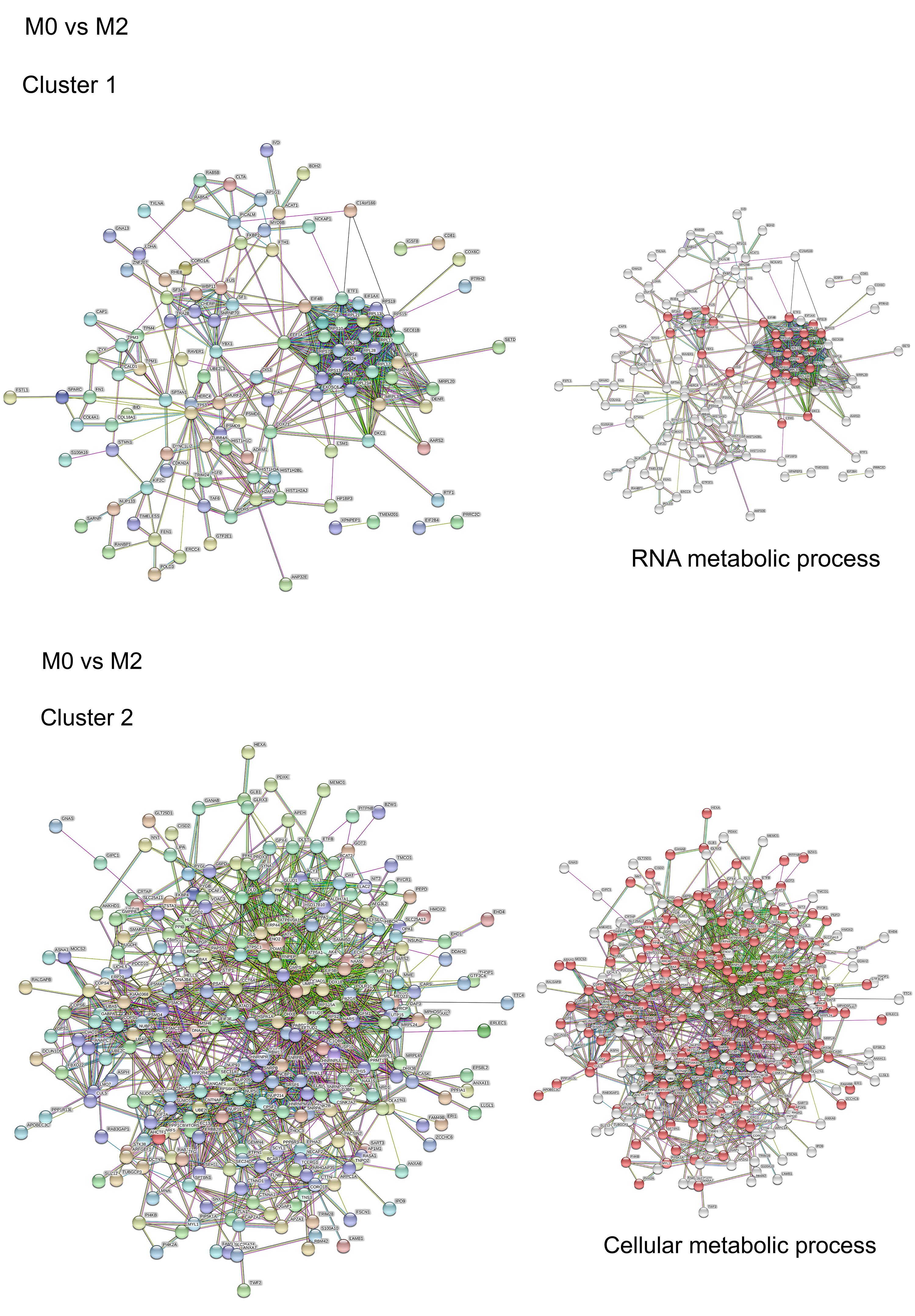

Supplement: Supplementary file 1 [file cells-08-00580-s001.zip › Supplementary Figures and Tables/Supplementary Figure 4.jpg]

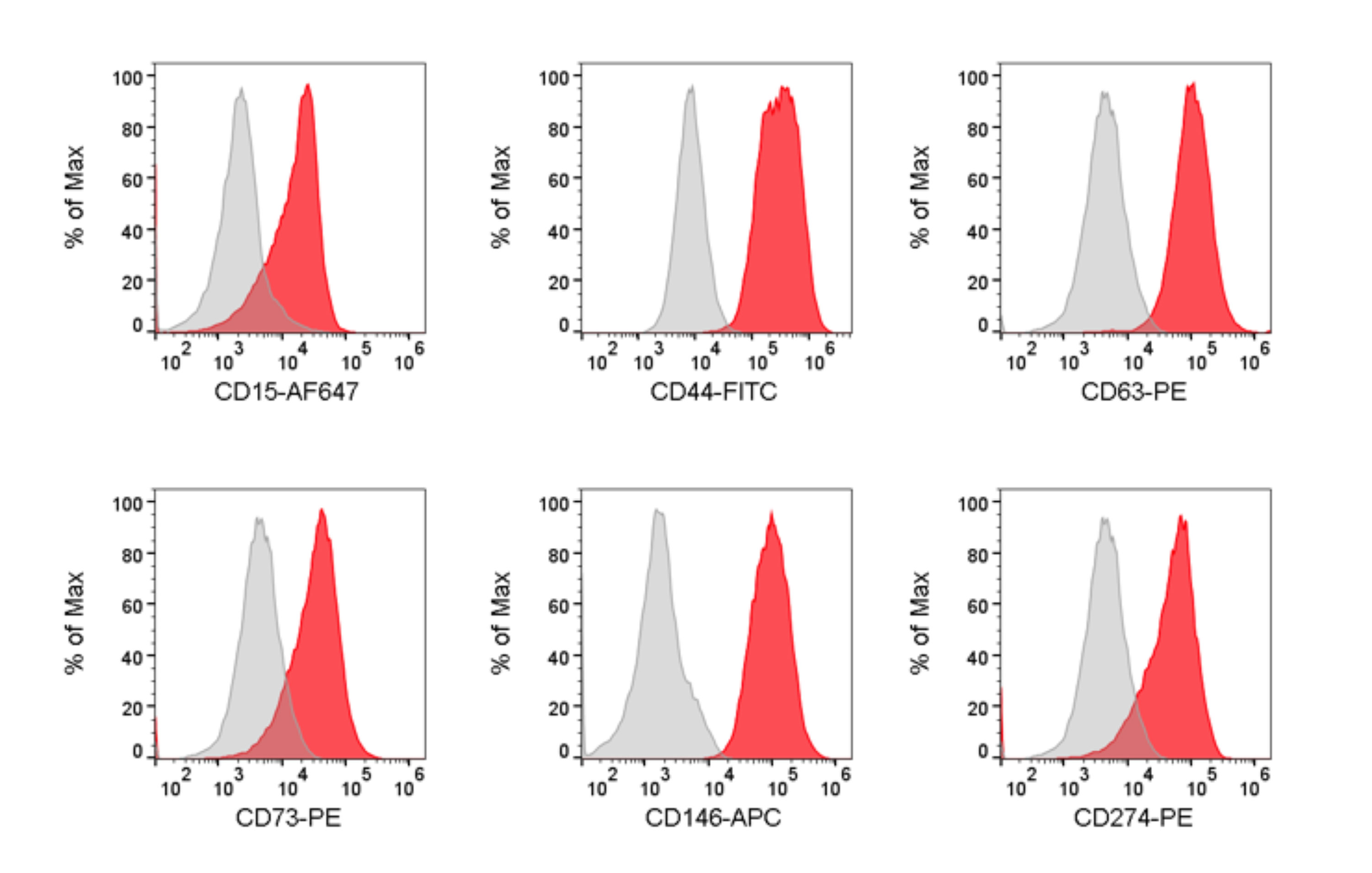

Supplement: Supplementary file 1 [file cells-08-00580-s001.zip › Supplementary Figures and Tables/Supplementary Figure 1.jpg]

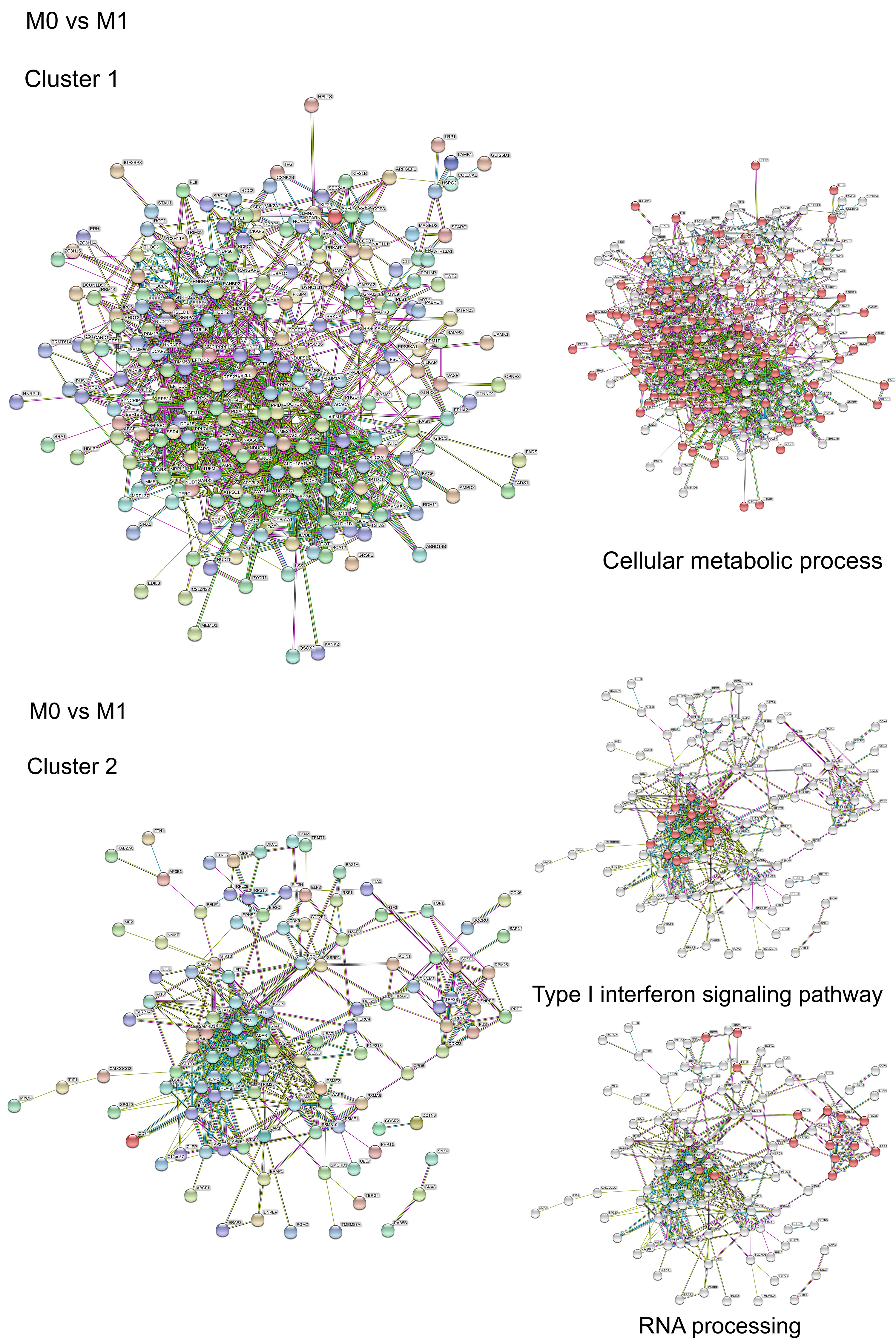

Supplement: Supplementary file 1 [file cells-08-00580-s001.zip › Supplementary Figures and Tables/Supplementary Figure 3.jpg]

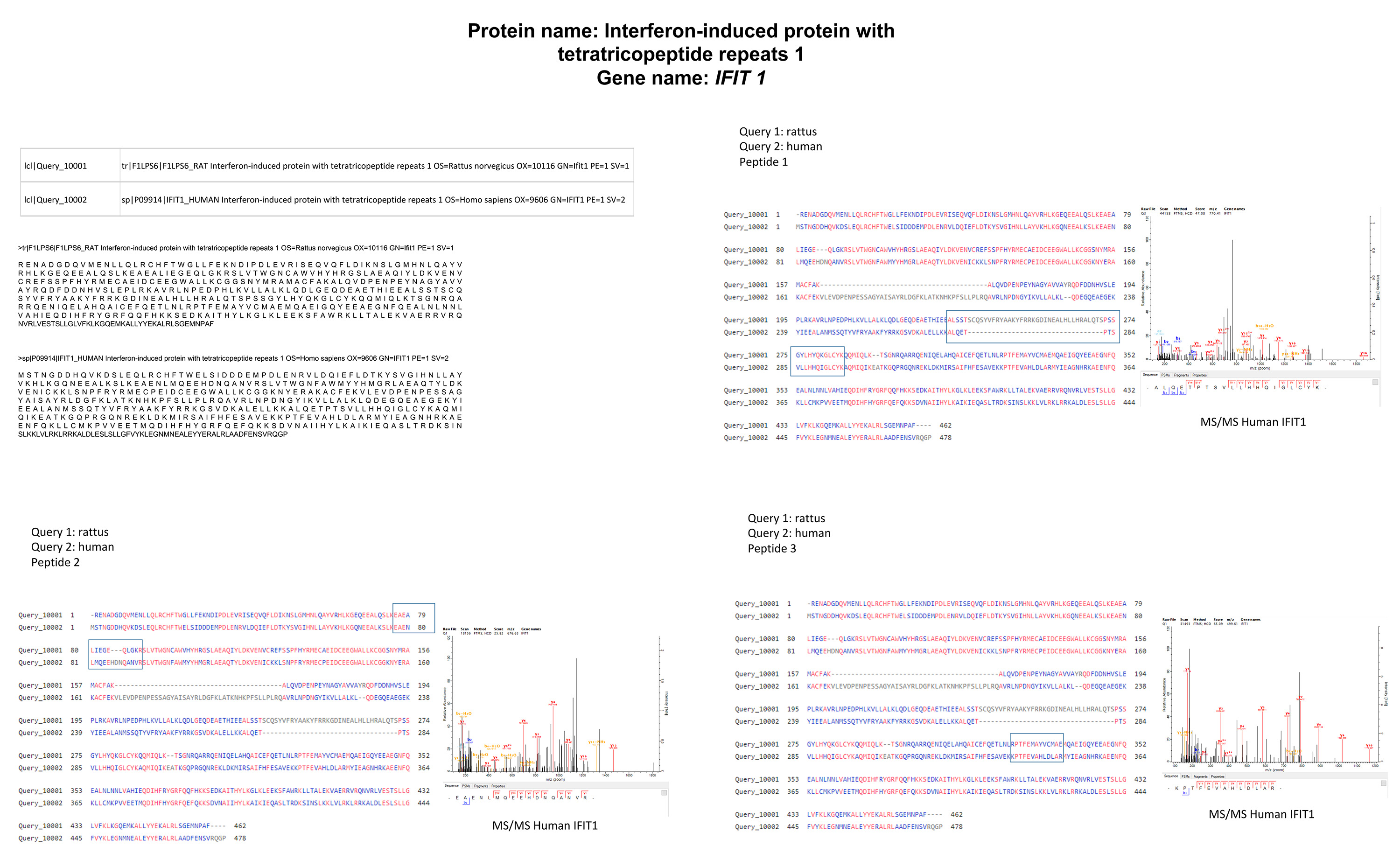

Supplement: Supplementary file 1 [file cells-08-00580-s001.zip › Supplementary Figures and Tables/Supplementary Figure 2.jpg]
